# Sensory reweighting for postural stability in individuals with low vision and blindness: balance adaptation and muscle co-contraction

**DOI:** 10.3389/fphys.2025.1684671

**Published:** 2025-11-14

**Authors:** Yue Li, Shilun Hou, Xin Zhang, Anli Wang

**Affiliations:** College of Sports Medicine and Rehabilitation, Beijing Sport University, Beijing, China

**Keywords:** posture control, sensory reweighting, visual impairment, muscle co-contraction, center of pressure

## Abstract

**Background:**

Individuals with visual impairments frequently experience postural instability during daily activities, considerably increasing the risk of falls. However, the mechanisms by which visually impaired individuals maintain balance through sensory reweighting remain unclear. We therefore aimed to investigate sensory reweighting for postural control in individuals with low vision and blindness by integrating measures of postural performance, biomechanical forces, and muscle co-contraction.

**Methods:**

Seventy-four participants were recruited (19 participants with normal vision, 36 participants with low vision, and 19 participants being blind). Each participant completed postural tasks under two conditions: open/closed eyes and firm/foam surfaces. Postural performance was evaluated with single-leg and tandem stance durations. The center of pressure (COP) during bipedal stance was collected using a force platform. Simultaneously, integrated EMG was acquired via wireless surface electromyography from six dominant-side muscles: erector spinae, rectus abdominis, rectus femoris, biceps femoris, tibialis anterior, and gastrocnemius.

**Results:**

We observed significant group × vision interactions for COP Path Length and Sway Area. The blind group exhibited the highest AP_HF% on a firm surface, confirming that individuals with visual impairment exhibit somatosensory compensation to maintain postural control. Individuals with low vision presented heightened sensitivity to partial sensory deprivation, with significantly increased Path Length and Sway Velocity. Additionally, a significant interaction between vision and somatosensation was observed, along with significant main effects of vision and somatosensation of all COP parameters. Muscle activity further supported these findings. The rectus abdominis/erector spinae ratio decreased significantly with somatosensory deprivation, whereas the gastrocnemius/tibialis anterior co-contraction index increased significantly under both visual and somatosensory deprivation, with higher co-contraction observed in both low-vision and blind participants.

**Conclusion:**

Blind individuals rely primarily on somatosensory input for sensory reweighting, while those with low vision show impaired compensation due to residual vision, resulting in the most impaired postural control. Ankle muscle co-contraction serves as the primary strategy for maintaining postural stability in visually impaired individuals.

## 1 Introduction

Postural stability refers to the ability of the body to maintain balance both during static positions and dynamic tasks (Lin et al., 2022). Nevertheless, individuals with visual impairments frequently experience instability during daily activities (Zarei et al., 2023; Pennell et al., 2024), which significantly increases the risk of falls and functional decline (Bhorade et al., 2021). Specifically, blind or partially sighted individuals are approximately 1.9 times more likely to fall than their sighted counterparts (Dhital et al., 2010). Therefore, the postural control underlying instability and fall prevention in this population remain a pressing challenge for public health.

The visual deprivation induces adaptive reorganization of sensory weighting for postural control in individuals with visual impairments (Chung and Barnett-Cowan, 2023; Zarei et al., 2025). Postural control relies on the central nervous system (CNS) to integrate sensory inputs from the visual, vestibular, and somatosensory systems and generate motor responses to maintain upright stability (Aubonnet et al., 2022). The absence of visual input in blind individuals compromises postural stability and necessitates compensatory reliance on proprioceptive and vestibular inputs through sensory reweighting (Parreira et al., 2023b; Helmich and Gemmerich, 2024; Bednarczuk et al., 2025). For instance, blind individuals demonstrate comparable stability to sighted individuals under eyes-closed conditions (Walicka-Cupryś et al., 2022; Parreira et al., 2023a), but exhibit reduced stability on unstable surfaces (Alghadir et al., 2019). This suggests that blind individuals primarily rely on somatosensory input to maintain postural control. Although this population demonstrates superior ankle proprioceptive acuity (Ozdemir et al., 2013) and non-visual sensory reweighting, their balance remains unstable, indicating insufficient and deficient compensation.

Additionally, residual vision in individuals with low vision may be associated with different sensory reweighting strategies (Chan et al., 2023). Compared to those with normal vision, individuals with low vision exhibit increased instability, such as more body sway, larger step width and slower gait during tandem walking (Tomomitsu et al., 2013). Within visually impaired populations, individuals with low vision demonstrate better static balance than those with total blindness (Bednarczuk et al., 2021). These studies suggest a linear relationship between the severity of visual impairment and postural instability (Bednarczuk et al., 2025). Nevertheless, some other studies highlight a nonlinear relationship contrasted to the aforementioned pattern. For example, under eyes-closed conditions, individuals with low vision were least stable in right single-leg stance (Bednarczuk et al., 2021). Also, another study reported that individuals with cataracts or retinal/optic nerve damage were less stable than those with normal vision or congenital blindness under eyes-open firm surface (Schwesig et al., 2011). These findings suggest that individuals with low vision remain highly dependent on visual input, and such reliance on residual vision may hinder the reweighting and compensation of other sensory modalities. Therefore, the combination of residual vision and non-visual sensory reweighting is not only insufficient to maintain postural stability in individuals with low vision, but may also represent a more severe deficit in sensory integration.

Given the sensory reweighting deficits observed, individuals with visual impairment may adopt an active stiffening strategy with muscle activation to maintain postural stability (Mohapatra et al., 2012; Dideriksen et al., 2015; Calalo et al., 2023). For example, a study on blind soccer players found that their adaptation to visual loss typically manifests as increased co-activation of the tibialis anterior and gastrocnemius muscles, thereby enhancing ankle stability and ensuring postural safety (Campayo-Piernas et al., 2017). Similar increases in muscle activation of ankle muscles have been observed in individuals with moderate myopia and in eyes-closed conditions (Huang and Xiao, 2022). These studies have quantified muscle activation magnitude during postural tasks. Specifically, muscle co-contraction, involving sagittal-plane activation of trunk and lower limb muscles, serves as a crucial mechanism for maintaining a stable upright posture (Cimadoro et al., 2013; Minamisawa et al., 2023). Therefore, the modulation of muscle co-contraction in postural control remains unclear. Additionally, the broader populations of individuals with blindness and low vision deserve further research attention (Zhang et al., 2024).

This study aims to investigate the sensory reweighting in postural control across varying severities of visual impairment, integrating postural performance, biomechanical forces, and muscle co-contraction. We hypothesize that: (1) individuals with visual impairment exhibit sensory reweighting, with increased reliance on somatosensory input, (2) individuals with low vision demonstrate reduced postural stability under visual deprivation, suggesting insufficient somatosensory compensation, (3) neuromuscular co-contraction is amplified in visually impaired individuals, particularly among those with low vision.

## 2 Materials and methods

### 2.1 Participants

We determined the sample size using G*Power 3.1. Assuming a medium–large effect size (f = 0.5), an alpha level of 0.05, and statistical power of 0.80, an analysis of variance indicated that a total sample size of 45 participants (15 participants per group) was required. Ultimately, we recruited 74 university students aged 18–25, including 19 individuals with normal vision (20.00 ± 0.82 years, 62.68 ± 14.91 kg, 1.68 ± 0.07 m), 36 participants with low vision (21.19 ± 2.08 years, 67.67 ± 15.39 kg, 1.71 ± 0.11 m), and 19 participants being completely blind (21.00 ± 2.33 years, 64.05 ± 10.39 kg, 1.68 ± 0.09 m). All groups exceeded the minimum required sample size, thus ensuring adequate statistical power. No significant differences in age, height, or weight were found across groups.

Prior to participation, all individuals reviewed and signed informed consent forms, thereby confirming their understanding of the study’s objectives and procedures. The study protocol received ethical approval from the Ethics Committee of Sports Science Experiments at Beijing Sport University (Approval No. 2023134H).

#### 2.1.1 Inclusion criteria

Participants were classified according to the World Health Organization guidelines and the 11th edition of the International Classification of Diseases (Dawson-Squibb et al., 2023). Visual acuity values are reported using the Snellen fraction. For reference, a Snellen fraction of 6/6 represents normal vision; 3/60 indicates very low vision, where a person can see at 3 m what a person with normal vision can see at 60 m.

i. Blind group: Defined as no light perception or light perception with visual acuity less than 3/60 in the better eye. Participants had congenital or early-onset total vision loss and demonstrated independent, proficient mobility in daily environments.
ii. Low vision group: Defined as visual acuity in the better eye equal to or greater than 3/60 but less than 6/18. Participants, including moderate and severe visual impairment but excluding mild impairment (Tomomitsu et al., 2013), had congenital or early-onset partial vision loss and demonstrated independent, proficient mobility in daily environments.
iii. Normal vision group: Defined as visual acuity in the better eye greater than 6/12 with no light perception issues.

#### 2.1.2 Exclusion criteria

Participants were excluded if they: (i) were currently enrolled in structured physical activity programs; (ii) had a history of musculoskeletal injuries or medical conditions affecting balance; (iii) were taking medications that affect the central nervous system, coordination, or balance; (iv) experienced vestibular symptoms (e.g., vertigo, dizziness); (v) had metabolic or neurological disorders, or signs of vestibular or peripheral neuropathy; (vi) had undergone surgery of the lower limbs or lumbar spine, or had any pathologies known to affect balance, foot sensitivity, or gait.

### 2.2 Procedure

The experimental protocol consisted of two stages: (i) postural performance was assessed in a field setting using standardized single-leg and tandem stance duration tests; (ii) biomechanical profiling was conducted in the laboratory using force platforms and surface electromyography (sEMG).

#### 2.2.1 Single-leg and tandem stance

Participants performed single-leg stances on both legs and tandem stances (heel-to-toe, non-dominant leg behind) under four conditions combining visual input (eyes open/closed) and somatosensory input (firm/foam surface) (Willis et al., 2013). All tests were conducted on a flat, non-slip surface in a randomized order. Each test was repeated three times and timed with a stopwatch by the same examiner. A 30-s familiarization period was provided prior to testing.

During single-leg stance, participants kept their arms relaxed at their sides while the non-supporting leg was lifted approximately 15 cm, maintaining about 20° of hip flexion and 45° of knee flexion. For tandem stance, participants aligned one foot directly in front of the other, maintaining a stable posture until balance was lost (e.g., foot shift or ground contact). The maximum trial duration was capped at 60 s.

#### 2.2.2 Static standing in the laboratory

Prior to testing, examiners explained all procedures in detail. To minimize learning effects, participants practiced each condition 3 times to ensure performance consistency. During testing, they wore tight-fitting shorts and short-sleeved shirts, and all performed the tasks in standardized athletic shoes to eliminate variability from personal footwear.

Participants completed static standing tasks on a force platform (Kistler 5695ADAQ, Switzerland) under systematically manipulated visual and somatosensory conditions. Muscle activity of the dominant leg was recorded using wireless surface electromyography (Delsys Trigno, United States) from the erector spinae, rectus abdominis, rectus femoris, biceps femoris (lateral head), tibialis anterior, and gastrocnemius (medial head). The dominant leg was determined as the specific leg that participants would normally use to kick a ball (Sadeghi et al., 2000; Alonso et al., 2011). Electrode placement followed guidelines from The ABC of EMG and SENIAM (Avdan et al., 2023).

Each trial was conducted in a natural double-leg stance and lasted 20 s (Nagai et al., 2011). Participants were instructed to remain as still as possible. Testing was performed under four randomized sensory conditions: (i) Eyes open on firm surface (baseline; all sensory inputs available); (ii) Eyes open on foam surface (somatosensory input altered, visual and vestibular inputs available); (iii) Eyes closed on firm surface (visual input removed, somatosensory and vestibular inputs available); (iv) Eyes closed on foam surface (visual input removed, somatosensory input altered, vestibular input available). In the eyes-closed condition, participants wore a lightproof eye mask to ensure complete visual occlusion. This approach minimized uncontrolled light perception and attention-related variability associated with voluntary eye closure.

In the eyes-open condition, participants focused on a fixed target at eye level to standardize visual input. A foam pad (60 × 50 × 8 cm) was placed on the platform to alter somatosensory feedback. Standing on a compliant surface has been shown to distort and delay plantar pressure distribution and ankle proprioceptive feedback, thereby reducing the reliability of somatosensory input without completely eliminating it (Willis et al., 2013). This foam-surface condition is therefore commonly used as an experimental manipulation of somatosensory disruption in postural control research. The order of conditions was randomized, and a 30-s seated rest period was provided between trials to allow adequate light adaptation when switching between visual conditions (Helmich and Gemmerich, 2024; Sugimoto et al., 2024). Participants could take additional rest if needed, and none reported any fatigue during testing. Participants with low vision and blindness were instructed not to wear any glasses or assistive visual devices during all tests to ensure consistent sensory conditions. All procedures were conducted by the same examiner to ensure consistency.

### 2.3 Data recording and analysis

Ground reaction force (GRFs) were collected using a three-dimensional force platform, embedded flush with the laboratory floor. Data were sampled at 1,000 Hz while participants maintained a natural double-leg stance on the platform for 20 s. To eliminate initial and terminal transients, the first and last 2 s of each trial were excluded, yielding a 16-s analysis window (from second 3–18). The GRF signals were then filtered using a fourth-order low-pass Butterworth filter with a cutoff frequency of 40 Hz. Based on the processed GRF signals, four center of pressure (COP) parameters were calculated to quantify postural stability: path length (mm), sway area (mm^2^), anteroposterior sway standard deviation (AP Sway SD, mm) and anteroposterior mean velocity (AP mean velocity, mm/s).

To further examine the effects of different sensory inputs on postural control, frequency-domain analysis was performed on COP_AP signals using Fast Fourier transform (FFT). The power spectra were divided into three frequency bands: low frequency (LF, 0–0.3 Hz), medium frequency (MF, 0.3–1 Hz), and high frequency (HF, 1–3 Hz). The LF, MF, and HF bands represent visual, vestibular, and proprioceptive contributions to postural control, respectively (Vieira et al., 2009; Kanekar et al., 2014). The power within each band was normalized to the total power across the three bands, expressed as a percentage, yielding frequency-domain indices (AP_LF%, AP_MF%, and AP_HF%).

To examine individual variability in sensory reweighting, we calculated frequency-domain parameters of COP in the anteroposterior direction (AP_LF%, AP_MF%, and AP_HF%). Based on the sensory contribution percentages during the baseline condition (eyes open on a firm surface), participants in each group were categorized into visual-, vestibular-, and somatosensory-dependent types. To explore the relationship between baseline sensory weighting and postural performance under sensory perturbation, paired scatterplots were generated using baseline frequency-domain ratios as predictors (X-axis) and COP parameters (AP sway SD or AP mean velocity) under baseline (Y_1_) and comparison (Y_2_) conditions as dependent variables. Linear regression analyses were performed, and the coefficients of determination (*R* ^2^) and significance levels (p-values) were reported.

Surface electromyographic (sEMG) signals were recorded using a wireless Delsys Trigno EMG system (Delsys, Trigno Wireless EMG, United States) at a sampling frequency of 2000 Hz. Raw signals were processed using Delsys EMGworks Analysis software. They underwent 4th-order Butterworth band-pass filtering (10–400 Hz) to reduce noise, followed by full-wave rectification.

Ground reaction force (GRF) and electromyography (EMG) data were analyzed using MATLAB 2020 (The MathWorks, Natick, MA, United States). To account for inter-subject variability due to factors such as skin temperature and electrode impedance, EMG amplitude normalization was performed separately for each muscle within each participant. Normalization referenced the maximum integrated electromyography (iEMG) value observed for that muscle across all experimental trials (Tankisi et al., 2020), enabling standardized comparison of relative muscle contraction across participants. An external synchronization protocol ensured temporal alignment of all recorded signals.

The flexor–extensor contraction ratio was calculated as the quotient of the normalized iEMG values of the agonist flexor muscles to those of the antagonist extensor muscles. Additionally, the co-contraction index (CCI) was computed for the trunk, knee, and ankle during the tests. The calculation method is shown in formula, where N denotes the number of samples within the time window, and “EMGlow” and “EMGhigh” correspond to the relative magnitudes of EMG signals from the two postural muscles at each sampling point (Nelson-Wong and Callaghan, 2010; McDonnell et al., 2025).

$$CCI=\int_{i=1}^{N}\;\frac{lowerEMG_{i}}{higherEMG_{i}}\times \left(lowerEMG_{i}+higherEMG_{i}\right)$$

### 2.4 Statistical analysis

Statistical analyses were performed with SPSS Statistics (version 26.0, IBM Corp). Normality of data distribution was assessed using the Shapiro-Wilk test, and homogeneity of variances was evaluated with Levene’s test.

A three-way mixed-design ANOVA was conducted to examine: (i) the three-way interaction among Group, Visual condition, and Somatosensory condition (Group × Visual × Somatosensory); (ii) two-way interactions: Group × Visual, Group × Somatosensory, and Visual × Somatosensory; (iii) main effects of Participant Group (normal vision, low vision, blind), Visual Condition (eyes open, eyes closed), and Surface (firm, foam).

Significant effects were followed by *post hoc* comparisons using Fisher’s LSD test with Bonferroni correction. Effect sizes were reported as partial eta squared ( $\eta_{p}^{2}$ ). Data meeting assumptions of normality and homogeneity of variance are presented as mean ± SD. Statistical significance was set at α = 0.05.

## 3 Results

### 3.1 Single-leg stance and tandem stance

**FIGURE 1:**
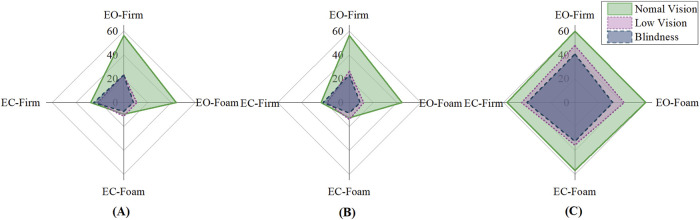
Single-leg and tandem stance duration of visual-somatosensory conditions. **(A)** Left leg stance Duration (s); **(B)** Right leg stance Duration (s); **(C)** Tandem stance Duration (s).

Figure 1 demonstrated the radar plot of standing duration across four conditions for each group, with larger areas indicating better postural performance. Significant group × vision interactions were found in left- and right-leg stance (p < 0.05), indicating that visual input influenced balance differently across groups (Suppl.1). The normal vision group relied heavily on visual input, as evidenced by significantly better performance under eyes-open conditions (p < 0.001) (Figure 1, top and right axes), whereas this advantage diminished under visual deprivation (Figure 1, left and bottom axes). The normal vision group demonstrated no difference from the visually impaired group under eyes-closed firm surface conditions (p > 0.05) (Figure 1, left axis). In contrast, visually impaired groups depended primarily on somatosensory inputs, demonstrating a significant decrease in standing duration on the foam surface (p < 0.001) (Figure 1, right and bottom axes).

### 3.2 Center of pressure (COP)

**FIGURE 2:**
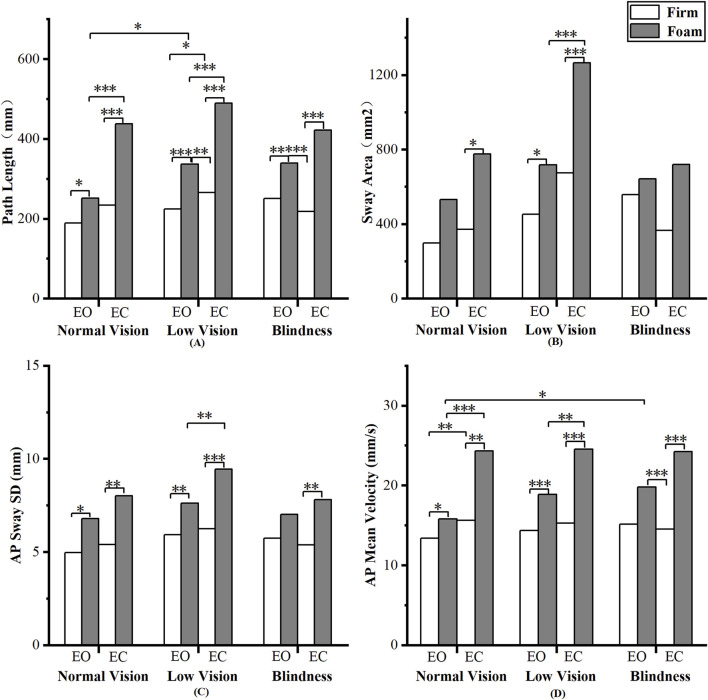
Pairwise analyses of visual-somatosensory effects on center of pressure (COP). **(A)** Path Length; **(B)** Sway Area; **(C)** AP Sway SD; **(D)** AP Mean Velocity.

We found a significant interaction between the visual × somatosensory (V × S, p < 0.05), as well as significant main effects of visual (p < 0.05) and somatosensory (p < 0.001) conditions on all COP parameters (Table 1). These results indicated that both visual and somatosensory deprivation independently impaired postural stability, and that their combined disturbance produced the most deterioration in balance performance. We also observed significant group × vision interactions (G × V, p < 0.05) in Path Length and Sway Area. Pairwise comparisons further revealed that, compared to the blind group, the low vision group was more sensitive to visual deprivation (Figures 2A,B).

**TABLE 1:** Main and interaction effects of visual-somatosensory conditions on center of pressure (COP).

Dependent variables	Group (G)	Visual (V)	Somatosensory (S)	G × V interaction	G × S interaction	V × S interaction	G × V × S interaction
Path length (mm)							
F value	1.624	28.014	79.523	2.974	0.446	22.387	0.13
P value	0.204	***p***_***0.001***_	***p***_***0.001***_	***0.048***	0.642	***p***_***0.001***_	0.878
$\eta_{p}^{2}$	0.044	0.283	0.528	0.077	0.012	0.24	0.004
Sway area (mm^2^)							
F value	142.051	4.745	19.092	3.337	0.76	5.6	0.186
P value	***p***_***0.001***_	***0.033***	***p***_***0.001***_	***0.041***	0.471	***0.021***	0.831
$\eta_{p}^{2}$	0.667	0.063	0.212	0.086	0.021	0.073	0.005
AP sway SD (mm)							
F value	2.773	7.393	40.853	0.993	0.288	5.888	0.231
P value	0.069	***0.008***	***p***_***0.001***_	0.376	0.751	***0.018***	0.794
$\eta_{p}^{2}$	0.073	0.096	0.369	0.028	0.008	0.078	0.007
AP mean velocity (mm/s)							
F value	0.381	31.453	67.311	2.200	0.358	21.214	0.164
P value	0.685	***p***_***0.001***_	***p***_***0.001***_	0.118	0.7	***p***_***0.001***_	0.849
$\eta_{p}^{2}$	0.011	0.310	0.490	0.059	0.01	0.233	0.005

*p*_*0.001*_ represent for p < 0.001. Bold values indicate statistical significance (p < 0.05).

Pairwise comparisons of COP parameters revealed the following patterns (Figure 2). First, a significant somatosensory effect was observed: nearly all COP parameters differed significantly between the Firm and Foam conditions (p < 0.05), indicating a consistent impact of somatosensory deprivation on postural control. Second, Path Length and AP Mean Velocity showed highly similar results, reflecting not only the significant effect of somatosensory deprivation but also a significant effect of visual deprivation on the normal vision and low vision groups (p < 0.05). Third, Sway Area and AP Sway SD were largely consistent, with the low vision group exhibiting heightened sensitivity to both visual and somatosensory deprivation.

To further investigate sensory weighting in postural control among individuals with visual impairment, we performed frequency-domain analysis on COP_AP signals using the fast Fourier transform (FFT) (Table 2). We found a significant interaction between visual and group across all three frequency bands (p < 0.05). This indicates that visual input affected COP frequency-domain characteristics differently across groups. We also observed a significant interaction between visual and somatosensory conditions for all indicators (p < 0.05), demonstrating that the two sensory modalities jointly influenced COP frequency-domain features. In addition, visual condition significantly affected all three frequency bands (p < 0.01), and somatosensory condition had a highly significant effect on all bands (p < 0.001). These findings demonstrate that both vision and somatosensation contribute to postural control across different frequency bands.

**TABLE 2:** Main and interaction effects of visual-somatosensory conditions on COP frequency domain characteristic.

		EO-firm	EO-foam	EC-firm	EC-foam		F value	P value	$\eta_{p}^{2}$
AP_LF%	Normal vision	62.07 ± 18.62	65.04 ± 18.34	59.19 ± 21.28	39.99 ± 21.03	Group	2.090	0.131	0.056
	Low vision	61.5 ± 18.86	46.93 ± 19.56	55.91 ± 20.43	36.53 ± 17.75	Visual	11.836	***0.001***	0.145
	Blindness	51.07 ± 21.24	44.08 ± 23.45	54.21 ± 18.35	45.01 ± 19.8	Somatosensory	21.923	***p***_***0.001***_	0.238
						G × V	5.005	***0.009***	0.125
						G × S	1.909	0.156	0.052
						V × S	6.058	***0.016***	0.08
						G × V × S	2.276	0.11	0.061
AP_MF%	Normal vision	32.65 ± 16.6	30.79 ± 17.17	32.58 ± 16.86	52.82 ± 19.79	Group	2.294	0.108	0.062
	Low vision	33.21 ± 16.7	46.62 ± 17.69	36.39 ± 16.48	57.97 ± 17.18	Visual	8.820	***0.004***	0.112
	Blindness	40.09 ± 17.13	49.49 ± 21.94	35.83 ± 13.38	49.33 ± 19.52	Somatosensory	34.671	***p***_***0.001***_	0.331
						G × V	4.151	***0.020***	0.106
						G × S	1.575	0.214	0.043
						V × S	11.316	***0.001***	0.139
						G × V × S	2.256	0.112	0.061
AP_HF%	Normal vision	4.67 ± 2.85	3.67 ± 1.93	7.44 ± 5.33	6.10 ± 2.63	Group	2.294	0.108	0.062
	Low vision	4.85 ± 3.8	5.58 ± 3.36	6.96 ± 6.06	4.72 ± 3.13	Visual	8.820	***0.004***	0.112
	Blindness	7.91 ± 5.93	5.63 ± 3.5	8.54 ± 6.09	5.00 ± 2.74	Somatosensory	34.671	***p***_***0.001***_	0.331
						G × V	4.151	***0.020***	0.106
						G × S	1.575	0.214	0.043
						V × S	11.316	***0.001***	0.139
						G × V × S	2.256	0.112	0.061

*p*_*0.001*_ represent for p < 0.001. Bold values indicate statistical significance (p < 0.05).

**FIGURE 3:**
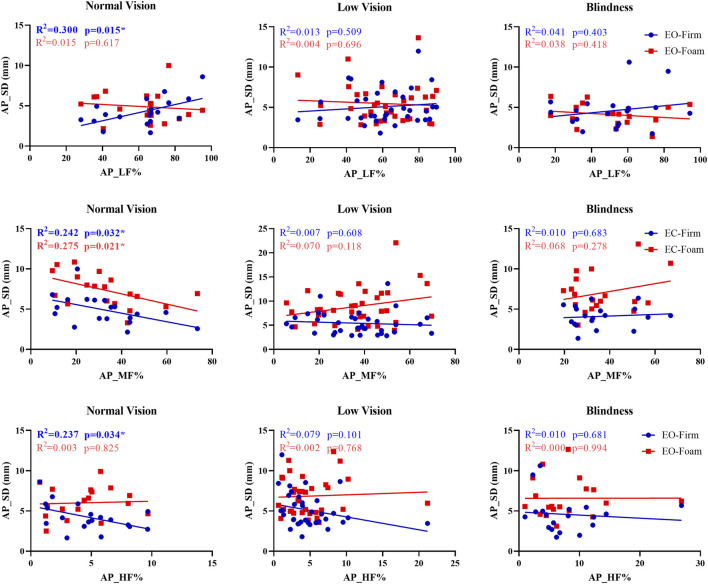
Relationship between sensory weighting and AP_SD.

Given the considerable individual variability in sensory reweighting, we examined the associations between sensory weighting and AP sway SD in three groups. In the normal-vision group, several significant associations were observed between sensory weighting and AP sway SD (Figure 3). Under the EO-Firm condition, higher low-frequency power (AP_LF%) was associated with increased AP sway SD (p = 0.015, *R* ^2^ = 0.300), indicating that visually dependent participants made more postural adjustments to maintain balance. Under the EC-Firm condition, medium-frequency power (AP_MF%) was negatively correlated with AP sway SD under both EC-Firm and EC-Foam conditions (p = 0.032, *R* ^2^ = 0.242; p = 0.021, *R* ^2^ = 0.275), suggesting that participants with stronger vestibular weighting maintained steadier posture when somatosensory input was perturbed. Additionally, higher high-frequency power (AP_HF%) under EO-Firm was associated with reduced AP sway SD (p = 0.034, *R* ^2^ = 0.237), indicating that stronger proprioceptive weighting was linked to more stable postural control on a firm surface.

**FIGURE 4:**
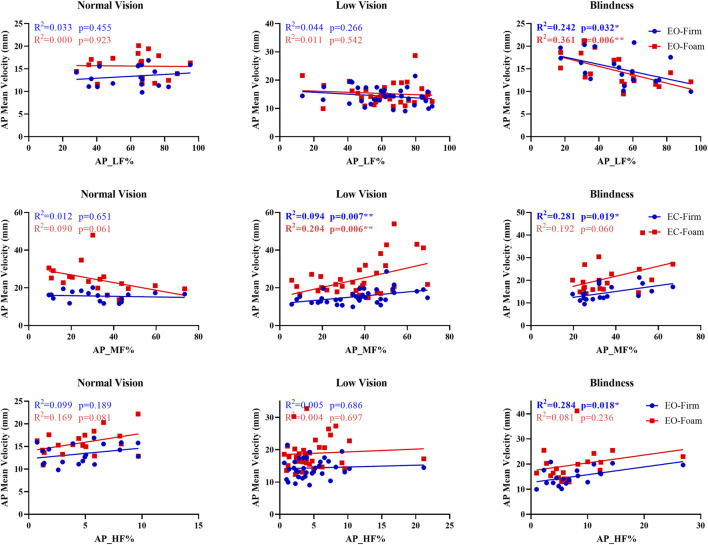
Relationship between sensory weighting and AP mean velocity.

Across the blind and low-vision groups, significant associations were found between sensory weighting and AP mean velocity (Figure 4). In the blind group, low-frequency power (AP_LF%) was negatively correlated with AP mean velocity under EO-Firm and EO-Foam conditions (p = 0.032, *R* ^2^ = 0.242; p = 0.006, *R* ^2^ = 0.361), indicating reduced reliance on visual input during static balance. Medium-frequency power (AP_MF%) under EC-Firm was positively associated with AP mean velocity (p = 0.019, *R* ^2^ = 0.281), and high-frequency power (AP_HF%) under EO-Firm was positively associated with AP mean velocity (p = 0.018, *R* ^2^ = 0.284), suggesting enhanced vestibular and proprioceptive weighting for refined postural control in the absence of vision. In the low-vision group, medium-frequency power (AP_MF%) demonstrated positive correlations with AP mean velocity under EC-Firm and EC-Foam conditions (p = 0.007, *R* ^2^ = 0.194; p = 0.006, *R* ^2^ = 0.204), indicating more reliance on vestibular input when visual cues were unavailable.

### 3.3 Muscle co-contraction

The RA/ES (F = 4.294, p = 0.042, $\eta_{p}^{2}$ = 0.058) and MG/TA (F = 3.878, p = 0.053, $\eta_{p}^{2}$ = 0.052) ratios were significantly influenced by the main effect of somatosensory input (Table 3), indicating that both the trunk and ankle are highly sensitive to somatosensory input from the surface. The MG/TA co-contraction index demonstrated significant increases under the main effects of vision (p < 0.001) and surface condition (p < 0.001), along with a significant group × vision interaction (p = 0.016) (Table 4). These results revealed that the ankle acted as a primary compensatory joint under sensory deprivation.

**TABLE 3:** Main and interaction effects of visual-somatosensory conditions on flexor-extensor muscle contraction intensity ratios (iEMG Ratio).

Dependent variables	Group (G)	Visual (V)	Somatosensory (S)	G × V interaction	G × S interaction	V × S interaction	G × V × S interaction
RA/ES							
F value	0.304	1.188	4.294	2.234	0.016	0.058	2.557
P value	0.739	0.279	***0.042***	0.115	0.984	0.81	0.085
$\eta_{p}^{2}$	0.009	0.017	0.058	0.06	0	0.001	0.068
BFlh/QF							
F value	1.916	0.136	0.396	0.982	0.056	1.869	0.887
P value	0.155	0.714	0.531	0.38	0.946	0.176	0.417
$\eta_{p}^{2}$	0.052	0.002	0.006	0.027	0.002	0.026	0.025
MG/TA							
F value	1.263	0.341	3.878	0.625	0.334	0.234	0.41
P value	0.289	0.561	***0.053***	0.538	0.717	0.63	0.665
$\eta_{p}^{2}$	0.035	0.005	0.052	0.018	0.009	0.003	0.012

RA, Rectus Abdominis; ES, Erector Spinae; BFlh, Biceps Femoris (Long Head); QF, Quadriceps Femoris; TA, Tibialis Anterior; MG, Medial Gastrocnemius. Bold values indicate statistical significance (p < 0.05).

**TABLE 4:** Main and interaction effects of visual-somatosensory conditions on CCI.

Dependent variables	Group (G)	Visual (V)	Somatosensory (S)	G × V interaction	G × S interaction	V × S interaction	G × V × S interaction
RA/ES							
F value	0.813	0.997	0	0.582	1.965	0.56	1.036
P value	0.448	0.321	0.986	0.561	0.148	0.457	0.36
$\eta_{p}^{2}$	0.023	0.014	0	0.016	0.053	0.008	0.029
BFlh/QF							
F value	0.358	0.014	1.195	0.269	0.41	0.711	0.237
P value	0.7	0.906	0.278	0.765	0.665	0.402	0.79
$\eta_{p}^{2}$	0.01	0	0.017	0.008	0.012	0.01	0.007
MG/TA							
F value	1.509	19.516	31.558	4.372	0.471	0.488	1.762
P value	0.228	***p***_***0.001***_	***p***_***0.001***_	***0.016***	0.626	0.487	0.179
$\eta_{p}^{2}$	0.041	0.218	0.311	0.111	0.013	0.007	0.048

*p*_*0.001*_ *represent for p < 0.001*. RA, Rectus Abdominis; ES, Erector Spinae; BFlh, Biceps Femoris (Long Head); QF, Quadriceps Femoris; TA, Tibialis Anterior; MG, Medial Gastrocnemius. Bold values indicate statistical significance (p < 0.05).

**FIGURE 5:**
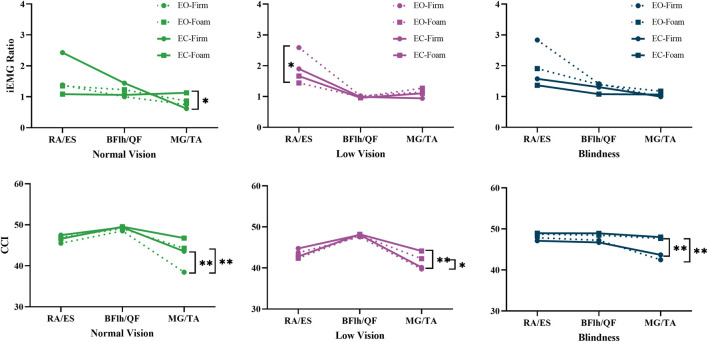
Pairwise analyses of visual-somatosensory effects on iEMG ratio and CCI.

Pairwise comparisons demonstrated that when either visual or somatosensory input was compromised, all three groups exhibited a reduction in the trunk flexor-to-extensor ratio (p = 0.032–0.09). Specifically, in the low-vision group, the RA/ES ratio significantly decreased from EO-Firm to EO-Foam (p = 0.032), indicating a substantial increase in extensor activation to prevent and control the slight forward-leaning tendency during upright stance (Figure 5, iEMG Ratio of Low vision).

The ankle CCI in the normal vision group was significantly influenced by both visual and surface conditions (p < 0.01), demonstrating higher values during eyes-closed and foam-surface standing compared with the eyes-open and firm-surface conditions (Figure 5, CCI of Normal Vision). In the visually impaired group, however, ankle CCI was mainly affected by surface conditions (p < 0.05), being higher on a foam surface under both visual conditions (Figure 5, CCI of Low Vision and Blindness).

In summary, the results demonstrate sensory reweighting for static postural control across three groups. Blind participants relied more heavily on somatosensory input when standing on a firm surface. The low-vision group showed reduced stability when visual input was partially unavailable, indicating incomplete somatosensory compensation. Neuromuscular co-contraction may contribute to changes in COP parameters, with the low-vision group exhibiting distinct ankle and trunk muscle co-contraction patterns. These findings indicate the adjustments in sensory and postural control across the severities of visual impairment.

## 4 Discussion

### 4.1 Sensory reweighting effects between groups

The present study confirmed that individuals with visual impairment exhibit somatosensory compensation in postural control. This was evidenced by the finding that, in the blindness group, single-leg stance duration increased and all COP parameters decreased from eyes-open to eyes-closed standing on a firm surface, suggesting that visual deprivation did not compromise postural stability when somatosensory feedback was available. In contrast, their stability declined substantially on a foam surface, indicating that disruption of somatosensory input markedly impaired balance. Furthermore, analysis of COP frequency-domain characteristics quantitatively confirmed this conclusion, demonstrating that the blind group exhibited the highest AP_HF% on a firm surface, indicating a predominant reliance on proprioceptive input for postural control. Consistent with previous studies (Gerber et al., 2024; Helmich and Gemmerich, 2024; Zarei et al., 2025), blind participants relied heavily on somatosensory and vestibular inputs, performing as well as or better than normal vision individuals during eyes-closed standing on a firm surface. However, their balance deteriorated more on a foam surface despite disruptions in all groups, consistent with neurophysiological evidence from Helmich (Helmich and Gemmerich, 2024) showing sustained hyperactivation of the sensorimotor cortex in blind individuals during postural tasks.

Low vision group presented a distinct sensory reweighting strategy. When visual or somatosensory inputs were compromised (eyes-closed or foam surface), participants demonstrated reduced single-leg stance duration and elevated COP parameters. This suggests that partial visual loss may hinder proprioceptive reweighting more than total blindness, possibly due to interference from residual visual input. This finding contrasts with Bednarczuk’s report of a linear increase in somatosensory dependence with impairment severity in adolescents (Bednarczuk et al., 2025). The difference is likely due to developmental factors, as our participants were young adults with stable motor skills, whereas adolescents are still acquiring them. The severity of partial vision loss may mark a critical threshold where vision is insufficient for stable balance, yet proprioceptive adaptation remains incomplete compared to total blindness.

### 4.2 Sensory reweighting effects within groups

The loss of either visual or somatosensory input alone undoubtedly leads to postural instability. Which is more important for postural control? In this study, we found that somatosensory loss has more impact on static balance than vision loss, and this effect was not limited to individuals with visual impairments. Among COP parameters, all groups exhibited reduced sway on the eyes-closed firm surface compared to the eyes-open foam surface (Figure 2). The main effect of somatosensory condition was all significant (p < 0.001) of COP parameters and COP frequency domain parameters, with very large effect sizes. For instance, the effect size of somatosensory deprivation on Path Length ( $\eta_{p}^{2}$ = 0.528) exceeded that of visual deprivation ( $\eta_{p}^{2}$ = 0.283). Muscle co-contraction strategies supported these findings. MG/TA CCI was mainly modulated by somatosensory input ( $\eta_{p}^{2}$ = 0.311), with visual input having a smaller effect ( $\eta_{p}^{2}$ = 0.218) (Table 4). RA/ES contraction ratio was highly sensitive to somatosensory feedback, but not to vision (Table 3). This finding appears inconsistent with some previous studies, which suggest that vision plays a dominant role in balance control (Ryan, 1940; Hutmacher, 2019; Liu et al., 2024). The static standing in our study and the simplified sensory input conditions in the laboratory may have contributed to this result. For instance, under eyes-open conditions, participants were instructed to fixate on a specific point straight ahead, which limited visual exploration. Previous studies have demonstrated that vision is essential for motor control in dynamic tasks, particularly in determining movement direction and coordinating interactions with the environment (Patla, 1997; Gautier et al., 2007; Krigolson et al., 2015). When somatosensory input is reliable, such as on a firm surface, vision primarily optimizes postural adjustments and perceive environmental cues (Goodale and Haffenden, 1998; Cullen, 2019; Petty, 2021).

The effect of visual deprivation on COP parameters revealed a different pattern. While the removal of visual input increased postural sway across all measures, the magnitude of change varied among parameters. Path Length and AP Mean Velocity showed the strongest main effects of visual deprivation (p < 0.001), whereas Sway Area and AP Amplitude exhibited smaller yet significant increases (p < 0.05). Path Length and AP Mean Velocity collectively reflect the dynamics and velocity of postural adjustments (Raymakers et al., 2005). Meanwhile, Sway Area and AP Sway SD primarily describe the spatial extent of body sway (Raymakers et al., 2005). In other words, the absence of vision alters the dynamics of balance regulation more than its spatial extent, implying that visual input mainly contributes to the fine-tuning of movement precision rather than gross stabilization during standing (Luo et al., 2025).

However, the relationship between corrective adjustments and sway spatial extent may not be synchronous, providing insights into the quality of postural control. For instance, when Path Length increases markedly while Sway Area and AP Sway SD increase only slightly or remain stable, it suggests that the body maintains sway within a confined spatial range by increasing the frequency of postural adjustments (Sozzi et al., 2023). This pattern represents effective and effortful control. Conversely, when all parameters rise substantially in tandem, it reflects a decline in postural control efficiency. This phenomenon was observed in the blind group. Under the EC-Firm condition, they exhibited a tendency toward longer Path Length compared with the sighted group, while Sway Area did not increase proportionally (Figure 2). This pattern aligns with the non-synchronous changes described above and suggests an active control strategy. It further implies that long-term visual deprivation may promote a more proprioception-dependent and fine-tuned postural control mechanism, allowing blind individuals to better constrain the spatial range of sway in the absence of visual input.

Contrary to the overall trend observed for somatosensory dominance, single-leg stance duration in individuals with normal vision did not follow the EO-Foam and EC-Firm conditions. Participants were able to maintain balance longer under the EO-Foam condition than under the EC-Firm condition (Figure 2). However, COP-based results indicated more postural sway under the EO-Foam condition, although the differences between conditions were not statistically significant (Figure 2). This finding suggests that individuals with normal vision can effectively utilize visual input to compensate for diminished somatosensory information and actively adjust their posture to maintain stability. Consequently, in this population, increased sway may not necessarily reflect impaired postural control but rather indicate a more flexible and adaptive postural strategy.

Dual sensory deprivation exerts a significant interactive effect on postural control across three groups, especially in the low vision group, leading to the most severe instability among all tested conditions. When both visual and somatosensory inputs are removed, individuals primarily rely on vestibular input to maintain balance (Karim et al., 2013). However, marked increases in all COP parameters indicate that vestibular input alone is insufficient for maintaining postural stability. These findings align with previous studies (Khadive et al., 2022; Lin et al., 2022; Zarei et al., 2025), demonstrating that the simultaneous loss of two sensory modalities exceeds the limits of compensatory capacity. Extending this insight, our results demonstrate that even individuals with normal vision have limited compensatory responses when postural demands are elevated, underscoring the task-dependent nature of sensory reweighting and the critical role of multisensory integration in maintaining balance.

### 4.3 Muscle co-contraction

This study further investigated neuromuscular co-contraction across four sensory conditions among severities of visual impairment. Under sensory deprivation, the ankle primarily compensates by increasing co-contraction (CCI), with strategies differing between groups: individuals with normal vision rely on visual feedback, whereas those with visual impairment depend on proprioceptive feedback. This is also consistent with the intergroup differences observed in COP results. This reweighting strategy may result from long-term cortical plasticity (Schieppati et al., 2014), which facilitates proprioceptive reweighting through enhanced muscle co-contraction without compromising postural stability. Proprioception provides afferent information on segment position and movement via receptors in joints, muscles, and tendons (Proske and Gandevia, 2012; Crevecoeur et al., 2016). In the upright stance, tactile cues from the feet, especially pressure information from the soles, play a crucial role in maintaining balance (Li et al., 2019). This is also consistent with Sugimoto’s (Sugimoto et al., 2024) observation that individuals with chronic ankle instability adopt environment-specific compensatory mechanisms that prioritize ankle strategies, underscoring the flexibility and adaptability of the neuromuscular system in response to sensory deficits. The low vision group adopts an ankle-dominant compensation strategy for postural control but demonstrates instability due to interference from residual vision. Clinically, emphasis should be placed on foam surface training to enhance proprioceptive reliance. Closing the eyes during tasks can reduce visual interference. Meanwhile, monitoring trunk compensation under eyes-closed firm surface conditions is important to prevent instability and falls (Pigeon et al., 2019; Taneda et al., 2021).

The ankle, thigh, and trunk extensor-flexor muscles contribute to static balance through distinct yet complementary mechanisms (Hill et al., 2023). The ankle muscles execute high-frequency, small-amplitude adjustments via dorsiflexion and plantarflexion (Donath et al., 2016). EMG analyses revealed that ankle muscle co-activation exhibited the most pronounced changes under sensory deprivation. These adjustments were accompanied by increases in COP Path Length and AP Sway Velocity, suggesting persistent corrective activity but with reduced efficiency. In contrast, the thigh muscles (quadriceps and hamstrings) primarily support knee extension and stability through co-contraction (Hirokawa et al., 1991). Their CCI remained largely invariant across conditions, confirming that knee locking is crucial for maintaining an upright stance. The trunk muscles (rectus abdominis and erector spinae) regulate anterior–posterior trunk tilt through coordinated activation to stabilize the center of mass in the sagittal plane (Claus et al., 2009). When this regulation is insufficient, the COP Sway Area increases. We found that standing on a foam surface induced a decrease in the RA/ES ratio in all three groups, with a statistically significant reduction particularly in the low-vision group. This reflects enhanced extensor activation as a compensatory strategy. Nevertheless, both COP Sway Area and AP Sway SD still increased, suggesting that this compensation may involve mechanical overcorrection, thereby exacerbating anterior–posterior instability.

Under somatosensory disruption, participants demonstrated increased COP parameters. This was accompanied by elevated co-contraction of the ankle muscles (MG/TA CCI), reflecting active distal-level postural adjustments to compensate for reduced proprioceptive feedback (Stoffregen and Bardy, 2014; Vette et al., 2017). At the same time, the RA/ES ratio decreased, reflecting more activation of extensor trunk muscles. This proximal adjustment likely stabilized the upper body and counterbalanced the instability caused by reduced ankle feedback. These findings suggest a coordinated proximal–distal postural control strategy, in which distal (ankle) muscles provide primary postural adjustments, and proximal (trunk) muscles contribute additional stabilization when distal control is insufficient (Shiratori and Latash, 2000). While COP alone cannot specify which muscles are active, combining COP and EMG results enables inferences of how postural control is distributed across distal and proximal segments.

### 4.4 Limitations

Although this study presented the sensory reweighting strategies in postural control across varying severities of visual impairment, three aspects still need further exploration. First, our participants were not stratified by the etiology or onset (congenital or acquired) of visual impairment, factors that may influence neural adaptations and compensatory mechanisms (Kupers and Ptito, 2014). Future research should consider stratifying subjects by etiology and onset to better understand their specific effects on sensorimotor adaptation. Second, we analyzed sagittal-plane antagonist muscle co-contraction using sEMG. Incorporating coronal-plane muscles and employing functional near-infrared spectroscopy (fNIRS) could provide a clearer understanding of medio-lateral COP dynamics and sensorimotor cortex activity (Helmich and Gemmerich, 2024). Third, the study tested only four sensory conditions. Real-life environments present more complex challenges such as unstable surfaces, multidirectional auditory inputs, and high attentional demands. In future research, we will investigate the effects of multisensory deprivation or overload on postural control in this population.

## 5 Conclusion

This study identified sensory reweighting and ankle muscle co-contraction as critical strategies for maintaining static postural stability in individuals with visual impairments. Low-vision individuals showed the highest instability under sensory deprivation, as their residual vision interfered with effective somatosensory compensation. These findings emphasize the importance of enhancing proprioceptive function and ankle neuromuscular control under low-visibility conditions to improve fall-prevention strategies. Future studies should further investigate the neurophysiology of these mechanisms and their contributions to dynamic postural control.

## Data Availability

The original contributions presented in the study are included in the article/Supplementary Material, further inquiries can be directed to the corresponding author.
